# Honouring the value of people in public health: a different kind of p-value

**DOI:** 10.2471/BLT.14.149369

**Published:** 2015-07-27

**Authors:** David Bishai, Abdul Ghaffar, Ed Kelley, Marie-Paule Kieny

**Affiliations:** a Interdepartmental Health Economics Program, Johns Hopkins School of Public Health, Baltimore, Maryland 21205, United States of America.; b Alliance for Health Policy and Systems Research, World Health Organization, Geneva, Switzerland.; c Service Delivery and Safety Department, World Health Organization, Geneva, Switzerland.; d Health Systems and Innovation, World Health Organization, Geneva, Switzerland.

When faced with a complex public health problem there is a natural urge to find solutions. We hire consultants, gather data, test hypotheses and examine *P*-values to identify risk factors: data-driven technological fixes get implemented every day. In the right situation, there is nothing wrong with solutionism – the belief that all difficulties have technical solutions. Solutionism works well for circumscribed problems involving a small number of motivated individuals, where every element of the prescribed solution can be implemented as planned. However, complex problems in public health usually have elements that defy planning, because health involves people, and people are unpredictable.

The World Health Organization recognizes that solutionism is unlikely to succeed in creating people-centred, integrated health systems.^1^ Public health problems are generated by communities, societies and cultures with their multiple histories, values and perspectives. This complexity leads to the predictable failures of solutionism: gleaming but abandoned tertiary hospitals, medical schools in poor countries pumping alumni to rich countries, bankrupt community insurance schemes and epidemics of lifestyle diseases checked only by pills and surgeries. Stymied solutionists say that the solutions they came up with were good, but the implementation failed.^2^ Rather than question solutionism’s unassailable worth, we blame the immature science of implementation.

The limits to solutionism become clear when coordinated actions of many people must be maintained over time. Public health problems are dynamic – they don’t stay solved. Action breeds an unpredictable reaction, requiring a repeat solution and the cycle continues. Dynamic problems don't have solutions so much as they have approaches.

Complex and dynamic public health problems require a different approach: an emphasis on the value of people. People who own the problem can anticipate the most likely social obstacles to its resolution, and their participation is essential to maintain an evolving strategy that can institutionalize an approach to the problem. Decades of community-based participatory research have shown that people from the community and their political leaders have to be included as full participants in problem solving.^3^ People are not just passive clients: they must be involved in deciding which problems need addressing, identifying the root causes and finding long-term solutions.

Solutionist approaches often feign deference to people by creating community advisory boards and holding occasional community briefings. This is not what we mean by honouring the value of people. Pro forma advisory boards are just a small step towards partnership: what is needed is a fundamental shift in the idea of who is in charge. Such a shift can be uncomfortable for technical experts who have spent years devoting themselves to a particular scientific orthodoxy and for whom the success of top-down solutions are testament to the solver’s goodness, worth and entitlement to deference.

Solutionism oddly dehumanizes the ones who need help: it spotlights the expert and marginalizes the persons closest to the problem. Communities get a subtle message that their insights are not valued. Contacting the community might be a concession because their survey responses can narrow down numerical parameters. With data in hand, the technician goes back to the *P*-value laboratory until emerging with the solution.

Many people view public health problems with different lenses and won’t necessarily accept an expert’s approach. Furthermore, local politicians who could have helped to identify obstacles are often excluded. We propose getting the community members, technical experts and politicians together from the start. They need to share their views about the system and commit to working together to solve problems.

Focusing on inclusion is not an appeal to folk wisdom. Community members are not experts in biomedical theory and are prone to set priorities based on personal anecdotes, fear and dread. These are serious weaknesses; however, technical experts can also have grave weaknesses in their ignorance of local social and cultural context. Not knowing how issues feel to participants is a real handicap for any solver – but those mutual weaknesses are why sustained partnership is needed. This principle of interdependence between technician and community, between clinician and family underpins a global movement that is committed to building people-centred health systems.

Recent research has shown that integrating community participation in the planning and implementation of health reforms is a key factor in supporting health improvements.^3^ The approach has been applied in a variety of areas including: the control of infectious disease;^4^ reducing maternal deaths and improved birth outcomes;^5^ enabling better health seeking behaviours;^6^ improving quality of life by promoting healthy environments through improvements to housing, reducing crime and building social cohesion.^7^ Critical factors for achieving trust include allowing participants to see their common concerns and building strong relationships within health committees or participatory groups. There must be a commitment to sustain long-lasting relationships between the community, local health workers and managers.^8^^,^^9^

Leading-edge programmes and public health activists are taking these principles forward all over the world and applying people-centeredness to both individual level and population level interventions, in both rich and poor countries. A project in Piedecuesta, Colombia, encouraged people with spinal cord injuries to meet regularly as a group to discuss their health-care needs. Health-care and social workers provided information on health and led interactive training sessions in practical self-care skills; topics covered included pressure sores, urinary problems, catheter management and sexual problems.^10^ In Nepal, engagement with local women’s groups identified major maternal and newborn problems in their communities. Community-driven strategies to address these had significant success with 30% fewer newborn deaths and 80% fewer maternal deaths.^11^ In India, participatory women’s groups met monthly in Orissa and Jharkand where a facilitator led them in participatory learning and action to identify and prioritize problems in maternal and newborn health. Neonatal mortality rates fell, especially among underprivileged groups. Service utilization trends remained the same among social groups.^5^^,^^12^^,^^13^

The way we think about community involvement is changing. Solutionists used to see community involvement as another intervention tool to be studied as though it were a bed net or a pill. If we value people, community involvement and engagement is not an instrument or an optional design feature for public health intervention: it is a non-negotiable commitment. There is no practice of public health without recognizing the value of people as partners and co-participants in the approach to commonly shared health problems.

Technical solutions for health problems are still needed. We still need the familiar *P-*value because biological evidence is necessary, but public health practice also needs to recognize the value of people. Regardless of the political environment, the power of the state to alter health decisions inside the home has limits. Only an approach that values, honours and engages people can alter how they make decisions about their health.
